# Recovering lost tax to meet the health financing gap for universal public sector health systems in East and Southern Africa

**DOI:** 10.1136/bmjgh-2023-011820

**Published:** 2023-10-09

**Authors:** Rene Loewenson, Chenai Mukumba

**Affiliations:** 1 Training and Research Support Centre, Harare, Zimbabwe; 2 Policy Research and Advocacy, Tax Justice Network Africa, Nairobi, Kenya

**Keywords:** Health systems, Health economics, Other study design

## Abstract

**Introduction:**

Universal healthcare services funded through taxation and free at point-of-access are the most equitable ways of funding healthcare rights. This paper examines key public sector health financing measures in 17 East and Southern African (ESA) countries, estimates the funding gap for basic and comprehensive services and relates this to sources of lost tax revenue.

**Methods:**

Health financing and tax data for 2018 (the most recent year available) were extracted from international databases for each ESA country, and analysed collectively for the region, comparing against intergovernmental estimates of optimal funding and tax capacity. Despite limitations noted, the scale of the health financing gap and tax losses informed policy recommendations.

**Results:**

The annual average per capita financing gap ranged from $28 to $84 for basic to comprehensive services, respectively, applying estimates of funding needs. Many innovative financing measures being explored do not meet this scale of deficit. Annual ESA per capita tax losses were estimated as: US$34.20 from shortfalls in domestic tax capacity and US$13.80 from illicit financial flows largely due to commercial practices. A proposed 25% minimum effective tax rate on multinationals in a fairer global tax system would yield an additional annual collection US$26.20 in the region.

**Conclusions:**

Addressing a total annual tax loss of US$34 billion from these three sources alone would almost completely finance the region’s US$36 billion financing gap for a comprehensive public sector health system. The COVID-19 pandemic’s exposure of the need for investment in public sector services suggests an opportunity for an alliance between health and finance sectors to ensure progressive taxation as the core funding for an equitable, universal health system. This implies costing the health funding demands and gap in ESA countries; strengthening domestic tax capacity, expanding wealth taxes, curbing illicit outflows and providing health evidence to ongoing African diplomacy for a fairer global tax system.

**WHAT IS ALREADY KNOWN ON THIS TOPIC**

- While direct taxation is documented to be the most progressive form of domestic resource mobilisation for universal health coverage, and comprehensive primary healthcare and a public sector health system to be critical for equity, East and Southern African (ESA) countries have faced challenges in meeting domestic and international commitments to adequate public financing for this and in overcoming catastrophic health spending.

**WHAT THIS STUDY ADDS**

- Comparing three areas of tax losses alone with shortfalls in public sector health financing in the ESA region, addressing a total annual tax loss of US$34 billion would come close to funding the US$36.8 billion shortfall in financing comprehensive public sector health systems in ESA countries.
- Addressing individual areas of tax loss from shortfalls on tax capacities, illicit flows due to commercial practices and unfair global tax rules could fund shortfalls in key public sector health services for universal health coverage and pandemic preparedness.

**HOW THIS STUDY MIGHT AFFECT RESEARCH, PRACTICE OR POLICY**

- Given the scale of the financing gap, beyond smaller pools of innovative financing currently being explored, the findings point to follow-up research and policy engagement to strengthen progressive taxation for public sector health system within the ESA region by addressing the identified areas of tax losses through building domestic capacity within revenue authorities, expanding wealth and other progressive taxes as substantial sources of revenue, blocking illicit financial flows and avoiding tax competition between ESA countries.
- Globally, the adoption of the December 2022 UN Resolution on International Tax Cooperation presents an opportunity to add health evidence to ongoing African diplomacy for a fairer global tax system, to meet public revenue from progressive taxation as the core source of funds for an equitable, universal health system and for pandemic and emergency preparedness in the region.

## Introduction

Funding health services from taxation, provided free at the point of access, is the most effective, equitable way of delivering public health services, including to deliver on the right to health.^1^ Despite this and the policy commitment to universal health coverage (UHC), neoliberal economic reforms have reduced public funding for social services and led to declining public health sector performance in East and Southern African (ESA) countries.^2^

Private sector proponents have argued that they can meet funding gaps for UHC, given inadequate domestic public sector funding and limitations on aid financing.^3 4^ Some ESA governments are contracting out primary care services to the private sector or expanding public–private partnerships to bring private financing into the health sector.^5^ There is some indication that the expansion of for-profit financing and service provision has been intensified by the demands of COVID-19.^6^

Relying on private sector initiatives for new resource flows to health services is, however, debated, with concerns over the implications for equity. Public–private partnerships are reported to incur substantial subsidies from public to private services, including in health worker out-migration from public to private services. A focus on curative personal care in for-profit services, given low profits from preventive care and cost barriers from fee charges are reported to undermine access and coverage to key services for low income communities.^4 5 7^

Such concerns have motivated advocacy for improved public sector health financing, that is, also a motivation for this paper, particularly if commitments to UHC are to be met and out of pocket (OOP) expenditure reduced.^1 8^ Beyond the global commitment to UHC, many ESA national constitutions include state duties to meet rights to healthcare, as also provided in the International Convention on Economic and Social Rights and General Comment 14, ratified by all ESA states, and in the African Commission on Human and People’s Rights.^9–11^ The underfunding of public services has thus led civil society to litigate rights claims or to demand accountability from public sectors to deliver on these rights and duties.^2 4^

While various options are being explored for improved public financing, we explore the role of taxes as the most equitable form of public financing.^1^ In particular, taking note of fiscal space constraints, and the positive role of fiscal capacity,^8^ we focus on addressing tax losses, particularly from tax avoidance, illicit financial flows (IFFs), tax waivers and other financial outflows.^12^ This is important, as research from South East Asia has shown that per capita government expenditure on health is positively influenced by fiscal capacity, as an indication of the overall size of the public sector in the economy.^8^ The level of tax collection as a share of gross domestic product (GDP) provides revenue for such public sector spending. While the link between tax revenue and the level of government health spending is not necessarily linear, some ESA countries have among the lowest tax to GDP ratios in the world, limiting their fiscal capacity and mobilisation of public revenue. For example, while Africa’s average tax to GDP ratio at 16% in 2020 was already much lower than the Organisation for Economic Co-operation and Development (OECD) average of 33.5% in that year, the tax to GDP ratio of Malawi was only 12.3%, of Botswana only 12.4%, notwithstanding its higher GDP, and of the Democratic Republic of Congo (DRC) only 7.3%, notwithstanding its significant natural resources.^13^

Given this context, we assess the size of the public sector health financing gap in 17 ESA countries, viz: Angola, Botswana, the DRC, Eswatini, Kenya, Lesotho, Madagascar, Malawi, Mozambique, Mauritius, Namibia, Seychelles, South Africa, Tanzania, Uganda, Zimbabwe and Zambia. We explore the potential for tax revenue to meet this gap, as the most progressive source of health financing for UHC, by addressing key areas of lost tax revenue. We, thus, suggest the implications to address these tax losses to improve tax funding to meet the public sector health financing gap, including for UHC.

ESA countries are economically diverse, with their per capita GDP in 2020 ranging (in US dollars) from Mozambique (US$449) and the DRC (US$557); to 10 times higher levels in Botswana (US$6711) and South Africa (US$5091). At the same time, some ESA countries with high GDPs also have high inequality, with the region’s highest Gini coefficient of 63% in South Africa, and coefficients of less than 45% in many ESA countries at lower GDP levels.^14^ Given this diversity, we present the evidence for the different ESA countries, while also providing aggregated and per capita estimates for the ESA region as a whole.

## Methods

For both health and tax data sets, we used global databases to allow for comparable evidence across ESA countries, costing levels in US dollars. For both health and tax data, with the work implemented in May to September 2022, 2018 was the most recent year available in intergovernmental databases at the time of doing the research. Where recommended US dollar levels were for years preceding 2018, they were adjusted for inflation to 2018 using the US$ inflation calculator.^15^

Evidence on key dimensions of public health financing was extracted by RL for 2018 for specific indicators for the seventeen ESA countries from the WHO Global Health Expenditure database.^16^ While there are multiple options for assessing public sector health financing, specific indicators of the adequacy and prioritisation of domestic financing used to inform the size of the financing gap were:

- The percentage of government spending allocated to the health sector, noting the Abuja Declaration commitment of 15% of domestic budget spending on the health sector.^17^
- The percentage of GDP spent on health, given that countries performing better in advancing towards UHC spend above 5% of their GDP on health.^18^
- The level of per capita public financing against estimates of recommended per capita health system funding derived from WHO^19 20^ and from health benefit calculations for selected ESA countries.^21^

We also assessed the share of private expenditure and of OOP spending as a percentage of total health expenditure in relation to public sector financing. The three estimates of recommended per capita funding in (c) above, adjusted for inflation to 2018 levels, were compared with the actual per capita spending for the ESA countries. This was used to identify the average funding gap for the ESA countries for each of the three recommended per capita levels. The total funding gap was assessed by multiplying the average per capita funding gap by the total population of the ESA countries.

The revenue raised by government from taxes was extracted by CM for 2018 for the seventeen ESA countries from online databases of the OECD, World Bank and Tax Justice Network. The tax measures included:

- Taxes from various sources as a share of total taxes.
- Tax to GDP ratios as a measure of tax capacity.
- Annual tax losses due to IFFs.
- Potential tax revenue gains from applying fairer international unitary taxation measures.

All health and tax data were captured in Excel spreadsheets by country, cross-validated by both authors and used to generate charts, tables and country and ESA aggregate estimates. The evidence and analysis was reviewed by external tax and health system expertise.

### Patient and public involvement

The work used only public domain secondary data sources for which ethical clearance was not required. Patients, patient advisors and the public were not involved in any way in the design, recruitment or conduct of the work reported in this paper and results of secondary data are being disseminated in public domain.

### Limitations

The selected indicators do not represent the full spectrum of measures of health financing and tax losses. For example, the level of pooling of different sources of financing enables income and risk cross-subsidies for equity and universality.^22 23^ Resource revenue data, fees and aid grants were not included, and their contribution to potential tax revenue merit further work. When we did the research (2021/2022) the most recent year in global databases preceded the pandemic period (2020–2022), when public spending on health systems increased significantly, although in particular areas of pandemic response, possibly leaving other areas with reduced funding.^14^ Within countries, national health accounts data could provide deeper evidence of sources of public health financing. Tax data may be drawn more directly from the African Tax Administration Forum (ATAF). However, we used the OECD, WHO and other intergovernmental databases for consistency, completeness and cross-country comparability.

Notwithstanding these limitations, the data provide sufficient evidence of the adequacy of public financing to assess the financing gap. The estimates of both health financing and tax losses are conservative, given higher spending demands for health security and rising non-communicable diseases (NCDs), and the likelihood of reported tax losses and outflows underestimating real levels. We recommend follow-up work using country data sources and national health accounts and including evidence on other taxes beyond those sources we examined. However, we consider the data applied to be sufficiently robust to justify the key policy messages drawn from the findings.

## Results

### Prioritising adequate public health financing

The commitment made in Abuja by heads of state to 15% of domestic government spending on health was triggered by the demands of HIV, tuberculosis and malaria.^17^ The demands from rising NCDs, epidemic outbreaks and pandemics, climate-related health shocks and the commitment to UHC suggest, however, that this commitment remains as relevant today as when it was made in 2001. While other sectors contribute to health, the Abuja commitment was regarded as a share that would reflect adequacy and prioritisation for the health sector to play its own role, including within whole of government approaches. Table 1 shows the level of current government health expenditure as a share of total government expenditure.

**Table 1:** Health expenditure data, east and southern African countries, 2018

Country	Domestic govt health exp as % total govt exp	Current health exp as a % of GDP	Domestic general govt health exp/capita in US$	Domestic private health exp as % current health exp	Out of pocket health exp as % current health exp
Angola	5.4	2.5	37	55	37
Botswana	14.3	5.8	374	16	3
DRC	4.5	3.3	3	50	42
Eswatini	6.0	6.5	89	25	24
Kenya	8.5	5.2	37	42	24
Lesotho	11.6	9.3	72	16	16
Madagascar	10.5	4.8	8	34	28
Malawi	9.8	9.3	10	18	11
Mauritius	10.0	5.8	282	56	49
Mozambique	5.6	8.2	9	16	10
Namibia	10.7	8.0	217	49	8
Seychelles	10.2	5.1	620	25	24
South Africa	13.3	8.3	284	44	8
UR Tanzania	9.4	3.6	16	25	24
Uganda	5.1	6.5	7	41	38
Zambia	7.0	4.9	30	16	10
Zimbabwe	7.6	4.7	39	52	24
Target	>15%	>5.0%	na	na	<15%

Source: World Health Organisation.^16^

DRC, Democratic Republic of Congo; exp, expenditure; GDP, gross domestic product; govt, government; na, not applicable; UR, United Republic.

In 2018, no ESA country had attained the 15% target, although some countries—Lesotho, South Africa and Botswana—were close to it. Seven ESA countries spent half or less than the 15% committed to by heads of state (table 1). A case study from Zambia suggested that the demands of the COVID-19 pandemic on an underfunded health system led to rapid mobilisation of funds above the Abuja commitment of 15% for health sector pandemic control, raising recognition of the need for investment in improved public sector health systems, including for future pandemic preparedness.^14^

### Adequacy of GDP shares and per capita public sector financing for health

Table 1 shows that spending on health exceeded 5% of GDP in 11 ESA countries in 2018, while noting that this combines resources from public, private and household sources. The evidence of public spending levels and OOP expenditure shares shown in table 1 suggest that fewer countries may have achieved this through significant public sector spending. Table 1 indicates the wide variability in per-capita government spending on public sector health services across the ESA region, with very low levels in six ESA countries, and countries with smaller populations and higher overall GDP appearing to fare better.

A number of estimates have been made of the funding needed for health system functioning. We used two WHO estimates and one estimate from country costings of essential health benefit packages to identify costs per capita for the public sector to deliver its health system obligations.

In 2012, WHO estimated that the minimum spending per person per year needed to provide basic, life-saving services was US$44.^19^ Adjusted for inflation, this translates to US$48/capita in 2018. As table 1 shows, 10 ESA countries spent below this 2018 level, with only countries with small populations and high GDPs spending above it. The average per capita funding gap for those spending below the basic threshold was US$28, ranging from US$9 in Zimbabwe to US$45 in the DRC.

In 2018, selected ESA governments calculated the costs for the public sector to deliver an essential health benefit.^21^ For the three countries that assessed total costs shown in table 2, the 2018 per capita cost ranged from US$52 to US$560, with the latter figure in Eswatini reflecting its small population as an outlier.

**Table 2:** Estimated US$ cost per capita for a public sector essential health benefit

Service level	Zambia		Uganda		Eswatini	
	US$/capita	Year	US$/capita	Year	US$/capita	Year
Total (including MoH and ancillary) in year shown	37.70*	2003	47.90	2012	519.00	2013
Total (including MoH and ancillary) in 2018†	51.47*	2018	52.41	2018	559.71	2018
Total (including MoH and ancillary) in 2022†	58.13*	2022	59.19	2022	632.08	2022

Source: Loewenson *et al.*^21^ All $ figures in US dollars based on conversion using exchange rate at year of costing.

*Including HIV interventions.

†The last two rows present the figure adjusted by inflation as calculated by the authors of this paper using the US$ inflation calculator.^15^

MoH, Ministry of Health.

For the majority of the countries in the region, it is reasonable to take the common US$52 found for the two other countries, one in east Africa and one in southern Africa, as a proxy. As shown in table 1, 10 ESA countries spent below this level, with an average per capita funding gap of US$32, ranging from US$13 in Zimbabwe to US$49 in the DRC.

These two prior estimates shown in table 2 are a conservative minimum. The 2000 World Health Report estimated that US$60/capita was needed for a comprehensive health system annually, including a minimal set of interventions and the infrastructure to deliver them. In 2001, this estimate was revised to US$80/capita per year.^20^ Adjusting for inflation, this translates to US$114/capita in 2018. As table 1 shows, 12 ESA countries with a combined population of 374.2 million^24^ spent below this level in 2018, with an average per capita funding gap in these countries of US$84, ranging from US$25 in Eswatini to US$111 in DRC.

Hence while five ESA countries—Namibia, Mauritius, South Africa, Botswana and Seychelles—funded their public sectors above all of these recommended per capita expenditure levels, the public sector per capita financing gap for the others ranged from an average per capita level of US$28 for the most conservative estimate of system needs, to US$84 for a more comprehensive system. For these 12 ESA countries, this implied a total shortfall in public financing that ranged from US$10.5 billion to US$31.4 billion. This represents a significant shortfall in public financing to deliver the minimum health service package, and even more so for a comprehensive health system.

### The contribution and implications of private and OOP health expenditures

Private financing comes primarily from OOP payments and voluntary private health insurance.^5 23^ Domestic private expenditure as a share of current expenditure varies widely across ESA countries, from 16% in Mozambique, Malawi, Botswana and Zambia, to over 50% in the DRC, Zimbabwe, Angola and Mauritius (table 1). The share of private expenditure rises not only when private financing rises, but also when public financing falls.

While this may point to a potential contribution from private financing to meet the public sector health financing gap noted above, a rising share of private financing coupled with falling public financing is also documented in the region to lead to outmigration of skilled personnel from the public sector, fragmentation of risk pools and a growth of power blocs resistant to regulation.^5 25^ In Uganda, for example, a decline in public financing and public health infrastructure triggered the emigration of many health workers, with a rising number of private sector services, including many illegal clinics and unregistered medical practices.^26–28^ Private voluntary insurance in ESA largely extends cover for higher income communities and formal sector workforces, with limited pro-poor benefit,^29^ while a focus on curative personal care in the for-profit sector over population health interventions is reported to exclude poor people and, particularly women, from positive health outcomes.^5^ While voluntary insurance includes some reimbursement of public and not-for-profit services in a number of ESA countries, only one country, South Africa, is currently working towards pooling tax and non-contributory and contributory financing in a national health insurance covering all members of the population.^29^

OOP spending below 15% of total health spending has been reported by WHO to reduce the possibilities of catastrophic expenditure, and the consequences for impoverishment and inequity.^30^ Table 1 shows that in 2018, 11 ESA countries exceeded this level of OOP spending on health. In part, this reflects rising private sector costs. In Kenya, for example, private sector prices were reported to have risen by 20% annually due to the collapse of agreements on pricing guidelines, with the introduction of new co-payments or a reduction in benefits used to manage cost escalation.^5^ While most ESA countries have policies of free services for particular levels or areas of public sector care, such as maternal and child healthcare, fee charges persist. When public funding does not cover the cost of adequate quality care in public services, informal charges may be levied in the public sector or households may shift to use more costly private services, contributing to rising OOP spending or a fall-out from health services, with negative health consequences, especially among lower income groups.^31–33^

While the specific implications of a rising share of private spending for health sector preparedness for COVID-19 would need further exploration, a regional review of responses to COVID-19 found the public sector role to be ‘critical and responsible in ensuring a co-ordinated, equitable and comprehensive response across all sectors, and for regulating and accrediting private sector activities.’(Chanda-Kapata, p2)^6^ The health financing evidence suggests therefore that efforts to mobilise additional private finances for health cannot substitute attention to ensuring adequate public sector health financing, particularly given the size of the financing gap noted earlier for even basic services.

### Taxes as a source of health system revenue

Governments collect revenue from taxes on income and profits, social security contributions and from other sources, including taxes on goods and services, payrolls and ownership and transfer of property. Tax revenues can play numerous functions, including redistributing income and wealth, repricing goods and services, and incentivising/disincentivising certain practices, including those that affect health. Tax systems also enable representation of various social groups on the basis of their rights as taxpayers.^34^ Direct taxes like income tax are levied directly on a taxpayer and adjusted to their characteristics. Indirect taxes are levied on transactions irrespective of the circumstances of the buyer or seller, such as on goods or services.^35^

**Figure 1:**
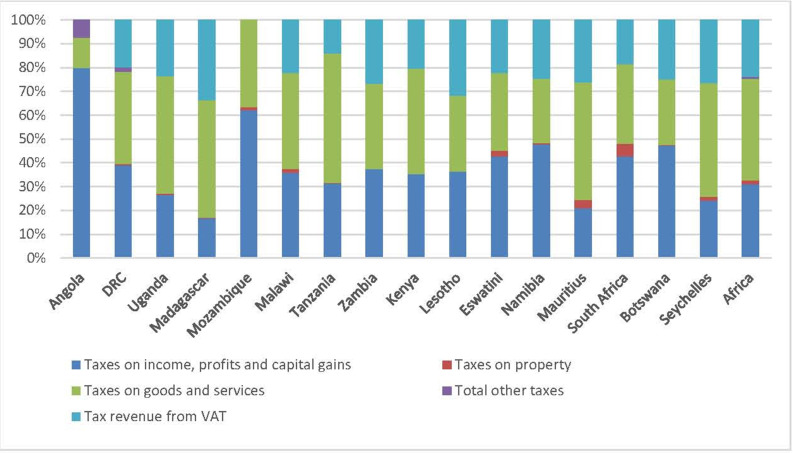
Different taxes as a percentage of total tax, ESA countries*, 2019. Source: authors from OECD.^36^ *Zimbabwe not included due to insufficient data. DRC, Democratic Republic of Congo; ESA, East and Southern African; VAT, value added tax.

The share of tax revenue from different sources for ESA countries is shown in figure 1. In 2018, taxes on goods and services, and taxes on income, profits and capital gains contributed similar shares (46%–47%). Value added tax (VAT) accounted for 27% of total tax revenue, higher than the 22% from taxes on income and the 15% from taxes on profits.^36^ While figure 1 indicates the variability between ESA countries, the generally lower share of taxes on corporate profits in the region suggests weak tax collection from this source, related in part to global tax rule and profit outflows, discussed later.

Beyond the total share of revenue, different sources of tax revenue need to address key health sector goals, including equity. Table 3 shows the progressiveness of the various sources of health financing for two ESA countries.^23^ It indicates that direct taxes are the most progressive source of revenue and can generate large funding pools, although they may be affected by economic downturns and be difficult to collect where informal employment is high. Health financing from indirect excise taxes, such as on tobacco and alcohol, is progressive if these goods are consumed to a greater degree by wealthier social groups, although in South Africa, excise taxes and the fuel levy were found to be regressive.^37^ As shown in table 3, private voluntary health insurance is a regressive form of taxation. Earmarking a surcharge on company or income tax, as applied in Zimbabwe’s ‘AIDS levy’, or a portion of VAT, as applied in Ghana to fund its national insurance, can add significant tax revenue for health.^29 38^ VAT contributes a significant amount to tax revenue in ESA countries, and may be a progressive source of funds for health where there is a large informal sector, provided it covers commodities consumed more by high-income groups, and if VAT collection thresholds protect small businesses.^29^

**Table 3:** Progressiveness* of different sources of health financing, ESA countries

Country	Total payments	Total public	General taxes	Direct taxes	Mandatory insurance	Indirect taxes	Total private	Voluntary insurance	Direct payments
Tanzania	+0.05	+0.18	+0.18	+0.48	+0.42	+0.07	−0.08	−0.49	−0.08
South Africa	+0.07	+0.01	+0.001	+0.04	na	−0.02	+0.06	+0.14	−0.04

Source: Authors using data from Mills *et al.*^23^

*Based on Kakwani indices for financing sources. A negative index shows a regressive mechanism and a positive index a progressive mechanism.

ESA, East and Southern African; na, not available.

### The tax to GDP ratio and tax gap

Despite its importance, the share of tax revenues in GDP in African countries of 16.6%, or the tax: GDP ratio, is one of the lowest in the world. It compares to averages of 21% in Asia Pacific, 22.9% in Latin America and the Caribbean and 33.8% in OECD countries.^13^ ESA countries have an average tax to GDP ratio of 18.1%.^13^ Tax to GDP ratios have increased by 1.8 percentage points over the past decade in Africa, primarily driven by increased VAT receipts and a growth in personal income taxes.^39^ Table 4 shows the wide variation between ESA countries in their tax to GDP ratio from below 10% in Angola and the DRC (countries with high levels of oil and mineral wealth), to ratios above 25% in South Africa, Seychelles, South Africa, Mozambique and Namibia.

**Table 4:** Tax data, east and southern African countries, 2018/2019

Country	Total annual tax loss, US$ (a)	Tax loss per capita (US$) (a)	Tax: GDP % 2019 (b)	US$/capita annual tax revenue gain using a weighted minimum effective tax rate of 25% (c)	Annual tax loss US$ millions from corporate base erosion and profit shifting, 2017 (a)	Tax loss/capita in US$ from corporate base erosion and profit shifting, 2017 (a)
Angola	361	11.7	9	12.9	361	11.7
Botswana	13	5.8	13	33.9	13	5.8
DRC	639	7.6	8	dna	639	7.6
Eswatini	15	13.2	18	14.1	15	13.2
Kenya	559	10.9	17	dna	559	10.9
Lesotho	1	0.5	21	55.4	1	0.5
Madagascar	77	2.9	11	0.4	77	2.9
Malawi	60	3.3	18	1.2	60	3.3
Mauritius	451	356.5	21	55.0	451	356.5
Mozambique	334	11.3	27	dna	334	11.3
Namibia	52	21.2	30	40.3	52	21.2
Seychelles	151	1556.7	34	21.7	151	1556.7
South Africa	3561	61.6	26	104.8	3561	61.6
UR Tanzania	213	3.8	12	1.1	213	3.8
Uganda	383	9	12	1.1	383	9
Zambia	635	36.6	17	7.1	635	36.6
Zimbabwe	107	7.4	14	3.5	107	7.4

Source: (a) Tax Justice Network^39^ (b) OECD^36^ (c) Cobham *et al.*^45^

dna, data not available; DRC, Democratic Republic of Congo; GDP, gross domestic product; UR, United Republic.

Each country has an optimum tax to GDP ratio, referred to as its tax capacity. The sub-Saharan African tax capacity is conservatively estimated to be 20%.^40^ The gap between this and actual tax capacities represents lost tax revenue. For the ESA region, the average tax capacity of 18%, yields an estimated tax gap of 2% of ESA GDP. With a GDP of US$773 billion in 2018, this indicates a lost tax revenue of US$15 billion, or a tax loss of US$34.2/capita for the 483 million people in the 17 ESA countries.^24^

Comparing the tax to GDP ratios in table 4 with the share of government spending on health in table 1 suggests that those countries with higher tax capacities also have higher shares of government spending on health. In Lesotho, Mauritius, Namibia, Seychelles and South Africa, tax capacities were greater than 20% and shares of government spending on health also exceeded 10%. There were outliers, although few, such as Mozambique, where tax was 27% GDP but the share of government spending on health only 5.6%, or conversely Botswana, with a lower tax capacity of 13% but a high share of government spending on health at 14.3% in that year.

### Losses to ESA tax revenue from global tax rules and IFFs

Several factors have contributed to the observed low tax to GDP ratios. The High Level Panel on IFFs from Africa reported poor governance, weak regulatory structures, harmful tax incentives and double taxation agreements as contributing to lower levels of tax collection.^41^

Tax losses can also be attributed to a global rule system that assigns tax collection to countries outside the region.

In a separate entity principle, multinational companies that operate under a common group and ownership can treat their branches in different countries as independent entities that work at arms-length. This enables companies to employ strategies to reduce their taxable income, termed ‘base erosion’ and to shift profits to other lower tax countries, both of which affect tax collection. In contrast, a unitary taxation approach allocates the overall profits of multinationals to the different states they operate in. Historically, the formula for country allocations has used the share of payroll costs, sales and physical assets, with continued benefit for high-income countries.^12^ In 2016, and in response to grievances raised around the inequity in the current global tax rules, the OECD proposed adapting the formula for tax liabilities for some multinationals to be applied on the basis of sales where final consumers are located. This, however, applies only to 25% of the profits of companies with revenues of more than US$21 billion and a profit margin above 10%, affecting less than 100 companies globally.^42^ Given the high volume of export of African commodities, it biases towards consumption in high-income countries.

Given this limited scope, African finance ministers have raised concerns that developed countries are not listening to the concerns of low/middle-income countries and are not significantly redressing an unfair balance in taxing rights.^43^ The demand for change towards a fairer global tax system became even more pressing given the significant costs generated by the COVID-19 pandemic and the increased deficits generated by the resultant trade and economic disruption.

Alternative approaches have been proposed. One proposal is for all countries to apply a minimum effective tax rate (METR) to avoid the incentive of shifting declared incomes to low tax countries or tax havens. A proposed METR of 25% is closer to Africa’s average corporation tax rate of 28%, given that an OECD proposal of 15% would still incentivise profit-shifting to OECD countries, given the higher rate in Africa.^44^

Table 4 shows how much ESA countries could gain by applying an METR of 25%. All ESA countries achieve some level of tax gain, and some, such as South Africa, Angola and Zambia, Lesotho and Mauritius, benefit more than others.^45^ Using the data in table 4, excluding the three ESA countries for which data were not available, applying a 25% METR in global tax rules would lead the region to gain US$7.2 billion in additional tax collection annually, or US$26.2/capita.

IFFs also contribute to losses in ESA tax revenue, as ‘financial flows that are illicit in origin, transfer or use; that reflect an exchange of value instead of a purely financial transactions; and that cross country borders’ (46:online) African countries are estimated to have lost more than US$50 billion annually over the past 50 years, exceeding the annual inflow of overseas development aid.^41^ An annual outflow of financial resources from the continent of US$88.6 billion reported in 2020 suggests that IFFs have increased.^47^ Most (65%) of these IFFs arise from commercial activities, while illegal activities and corruption account for 30% and 5% of IFFs, respectively.^41^ Commercial IFFs stem from transfer pricing, trade mispricing, misinvoicing of services and intangibles and use of unequal contracts, enabling tax evasion (illegal), aggressive tax avoidance (legal) and illegal export of foreign exchange.^41^ Legal tax avoidance is also enabled by the global rules system noted earlier, in providing legal loopholes to minimise tax liabilities in African countries.^41^

Depending on the estimate, base erosions and profit shifting to tax havens alone collectively cost low-income countries between US$167 and US$200 billion annually in lost corporate tax revenue.^48^ Table 4 shows the estimated losses for ESA countries, drawing on 2017 data.^39^ The evidence suggests that the ESA region lost US$7.6 billion annually in tax revenue due to global practices of base erosion and profit shifting, or US$124.7/capita, equivalent to 1.6% of the region’s GDP. Given that Seychelles and Mauritius with their low populations contribute to high per capita averages, excluding these two countries suggests an average annual per capita loss from these two commercial tax practices alone of US$13.8. This is a conservative estimate as it excludes losses due to other IFF sources and losses due to limited taxation of natural resource depletion of extractive activities.

## Discussion

### Relating health budget deficits to tax losses

ESA countries are exploring various forms of innovative financing. The additional funds they bring can, however, be small relative to the scale of demand, and equity and universality call for the core of the public sector health system to be funded from taxation or mandatory national insurance. The public sector health financing gap can thus be related to lost tax revenue due to shortfalls in revenue vs tax capacity, and tax losses due to current global rules and IFFs.

Table 5 consolidates the evidence found on the public sector health financing gap and the lost tax revenue, per capita and as totals. The combined tax losses from various sources total as an annual tax loss to the region of US$34.1 billion, a total tax loss that could come close to financing the US$36.8 billion shortfall in public sector health financing for a comprehensive health system in the ESA region, noted earlier. Even individual areas of tax loss, particularly from shortfalls on tax capacities and unfair global tax rules could fund shortfalls in public sector health financing of essential or basic services.

**Table 5:** Annual total and per capita US$ public sector financing gap and lost tax revenue, 2019

Area	For ESA countries		Year of data	Source
	US$ total billion	US$ /capita		
Public health financing gap				
Cost of an essential health benefit in ESA	14.0	32.0	2018	19
Minimum annual spending to provide basic, life-saving services	12.3	28.0	2012	17
Comprehensive health system, including a minimally adequate set of interventions and the infrastructure to deliver them*	36.8	84.0	2018	18
Taxes lost to public revenue				
Tax losses due to the shortfall between tax capacity and actual collections	15.0	34.2	2019	40
Tax losses due to non-application of a unitary taxation METR†	11.5	26.2	2016	45
Tax losses from global tax abuse from base erosion and profit shifting	7.6	17.4	2017	39

*Original year figure adjusted by inflation using the US$ inflation calculator,^15^ and applies the total population to the per capita figure.

†Per capita figure estimate excluded the DRC, Kenya and Mozambique but applied in this table to the total ESA population to estimate total tax loss to the region.

DRC, Democratic Republic of Congo; ESA, East and Southern African; METR, minimum effective tax rate.

While the limitations of these estimates have been noted, the scale of the figures suggest the significant contribution that addressing tax losses can make to meeting the gap in public sector financing of health services, to meet commitments to UHC, address demands for health security and emergency or pandemic preparedness, and to provide financial protection and comprehensive primary healthcare to address inequities in health. Many tax losses noted in the findings arise from taxes on wealth or relate to taxes being fairly collected within, rather than outside, ESA countries. This suggests that addressing these losses will not burden poorer populations. Engaging with these sources of lost tax thus presents an important avenue for equitable domestic health financing.

### Implications for meeting health financing gaps

Meeting the health financing gap in ESA countries calls for clarity on the funding demands ESA countries face to deliver the right to healthcare and commitments to UHC and equity, and to prevent and manage demands from rising NCDs, pandemics and climate change. The call for a public sector health system that is domestically financed above 5% of GDP and 15% of government budgets remains pertinent, with steps to pool tax funding with other health financing sources to overcome segmentation, enable risk and income cross subsidies, and to ensure that OOP spending does not exceed catastrophic health spending levels. The costing of public sector health services and the system infrastructure required for this, and the deficit on current financing need to be made clear and widely shared in public domain and with parliamentarians, with the understanding that this is most sustainably, equitably and adequately met through progressive taxation.

While this paper has focused on the overall levels of health financing, as is well noted in separate literature, the allocation of health resources, the level of spending on primary care and comprehensive primary healthcare services and on key social determinants of health are equally important for achieving better and more equitable health outcomes. At the same time, and as an area for separate inquiry, there is some indication that falling levels of overall public spending on health appears to weaken domestic spending on these key areas.^14 21 25 29 37^

Strengthening progressive taxation for health implies redoubling efforts at national level to address the tax gap by building domestic capacity within revenue authorities, by expanding the tax base through expansion of wealth and other progressive taxes as substantial sources of revenue, and by increasing transparency in and blocking of IFFs, such as through beneficial ownership transparency registries. It calls for work with the ATAF to avoid tax competition between ESA countries, and to reduce the tax incentives and exemptions for corporates that reduce ESA country capacities to mobilise tax revenue.

The global engagement by African countries on the Agreement on Trade-Related Aspects of Intellectual Property Rights waiver, backed by health diplomacy, as well as African finance ministers’ engagement on the global tax system indicate the potential when trade, health sectors, professionals and civil society converge on regional interests, including in challenging inequities in global systems that undermine responses within the region. The OECD Inclusive Framework platform discussing proposed reforms to global tax rules and formulae excludes many African countries. Seven ESA countries—Uganda, Mozambique, Zimbabwe, Tanzania, Malawi, Madagascar and Lesotho—are not members of the platform. Even those that are members do not participate at the same level as OECD member states. The unanimous adoption in December 2022 by the UN of a resolution on promotion of inclusive and effective international tax co-operation, promoted by African countries, thus opens the possibility for a more inclusive and transparent global architecture to set fairer global tax rules and for negotiation of a UN tax convention.^49^

Given its focus on specific areas of tax losses, further inquiry is merited on other public revenue sources, such as from other IFF flows or from taxation of natural resource depletion of extractive activities. Addressing these revenue losses may also finance national goals and sectors that contribute to the social determinants of health. Further, even if not all the funds raised by addressing the tax losses indicated in this paper are allocated to meet the public sector health financing gap for UHC, evidence suggests that the improved fiscal capacity from addressing these tax losses positively influences government spending on health.^8^

## Conclusions

As resources for the health sector have fluctuated or fallen, the interaction between health and finance ministries has often focused on improved efficiency and accountable use of funds, both of which are relevant concerns. Yet the financing gap for health systems, exposed by the challenges faced in meeting core services, in reducing OOP spending and managing the additional demands during the COVID-19 pandemic, together with the reliance in many countries on unpredictable external funding for core health services, all indicate that it is equally critical to address issues of adequacy and equity. This implies shifting the dialogue between health and finance sectors to a joint discussion on what needs to be done to ensure adequate public revenue. This dialogue has commenced with the interactions on smaller pools of innovative tax financing, but with a large and increasing unmet demand and the current health financing gap, it is timely to deepen this to joint advocacy on the tax gap and tax losses.

The evidence in this paper indicates that it is possible for adequate funding from progressive taxation to contribute to meeting the health financing gap for ESA public sector health systems. There are measures that ESA countries can take to address tax losses and to use public revenues gained to strengthen public sector health systems to meet constitutional commitments to healthcare and to UHC. While the tax revenues gained may also be shared with other sectors that play a role in health, the evidence points to the positive influence that the resulting improved fiscal capacity has on public sector health expenditure. Closing loopholes within the tax system provides an important basis for increased government spending on health.

The adoption of the December 2022 UN resolution promoted by African states to reform the global tax architecture under the UN adds a further important opportunity to include health evidence to support the case made by African finance ministers for a fairer global tax system. The health sector shows both need and opportunity to improve public revenue by applying fairer tax measures in a more inclusive global mechanism, and through greater domestic capacity, to ensure progressive taxation as the core source of funds for an equitable, universal health system in the ESA region.

## Data Availability

Data are available in a public, open access repository. The work used only public domain secondary data sources.
